# Muriate of Opium

**Published:** 1849-03

**Authors:** 


*Muriate of Opium.*—Dr. Nichol, in the Pharmaceutical Journal, says:

I prepared solutions of opium with acetic, nitric, sulphuric, citric, tartaric, and muriatic acids, and also prescribed them, but the muriatic solution was vastly superior to any in every respect. Each of them produced headache with the exception of the muriatic. I prefer muriate of opium to the tincture, wine, or powder of opium, and also to the muriate and acetate of morphia; in fact, to any other preparation of opium. It never causes headache. My preparation is made according to the following formula:—The best powdered opium, one ounce; muriatic acid, one ounce; distilled water, twenty ounces; mix. Shake this mixture very frequently every day, during fourteen days, then strain and filter. The dose is from twenty to forty drops.—*Ibid.*

